# Anaerobic Biodegradation Tests of Poly(lactic acid) under Mesophilic and Thermophilic Conditions Using a New Evaluation System for Methane Fermentation in Anaerobic Sludge

**DOI:** 10.3390/ijms10093824

**Published:** 2009-09-02

**Authors:** Hisaaki Yagi, Fumi Ninomiya, Masahiro Funabashi, Masao Kunioka

**Affiliations:** National Institute of Advanced Industrial Science and Technology (AIST), Higashi 1-1-1, Tsukuba, Ibaraki 305-8565, Japan; E-Mails:h.yagi@aist.go.jp (H.Y.);f.ninomiya@aist.go.jp (F.N.);m.funabashi@aist.go.jp (M.F.)

**Keywords:** anaerobic biodegradation, poly (lactic acid), methane fermentation

## Abstract

Anaerobic biodegradation tests of poly(lactic acid) (PLA) powder were done at the thermophilic (55 °C) and mesophilic temperature (35 °C) under aquatic conditions [total solid concentrations of the used sludge were 2.07% (at 55 °C) and 2.24% (at 35 °C)] using a newly developed evaluation system. With this system, the evolved biogas is collected in a gas sampling bag at atmospheric pressure. This method is more convenient than using a pressure transducer or inverted graduated cylinder submerged in water. PLA was degraded about 60% in 30 days, about 80% in 40 days and about 90% in 60 days at 55 °C. On the other hand, the PLA degradation started in 55 days at 35 °C and degradation rate was much slower than at 55 °C.

## 1. Introduction

Anaerobic fermentation has some advantages when compared to aerobic fermentation, such as composting. The anaerobic fermentation plant is a nearly closed system compared to the more stenchful aerobic one with a shorter processing time, and produces CH_4_ as an energy source [9–11]. In anaerobic fermentation plants, the non-biodegradable garbage collection bags are currently separated from feed stocks of anaerobic fermentation. The product, such as a garbage collection bag, made with biodegradable plastics and its waste is thought to be anaerobically degraded with household organic waste or animal manure in anaerobic fermentation plant. To verify whether biodegradable plastics are degraded in an anaerobic fermentation plant, their biodegradability must be measured in a laboratory, preferably by standard test methods.

Two International Standards (ISO) methods (ISO 14853, 15985) and several American Society for Testing and Materials (ASTM) methods (ASTM D5210, D5526, etc.) have been published as the international standard test methods for evaluating the anaerobic biodegradation of plastics. Table 1 lists the test conditions of these methods. ISO 14853 and the equivalent ASTM D5210 are aquatic biodegradation tests (TS 0.1–0.3% or >0.1%) at a mesophilic temperature (about 35 °C) in a synthetic growth medium with a mixed microbial population derived from a compost or waste water treatment facility. In these tests, the sludge is diluted with a mineral salts medium, so only microorganisms utilizing the polymer as a carbon source in the mineral salts medium grow. The microbial population is thought not to reflect the original sludge. ISO 15985 and ASTM D5526 are tests using over a 20% TS concentration (a high-solid condition) sludge at a thermophilic temperature (about 52 °C in ISO 15985) or mesophilic temperature (about 35 °C in ASTM D5526) with mixed inoculums derived from anaerobic digesters operating only on pretreated household waste.

Thermophilic (about 55 °C) anaerobic fermentation plants using a garbage slurry or animal manure as the feed stock, in which total solids concentration is about 10%, have recently been built and are operating in Japan. The operating conditions in these plants are different from the test conditions of the present ISO and ASTM methods. Therefore, bioplastics degradability in these thermophilic anaerobic fermentation plants are thought not to be measurable by the present ISO or ASTM methods.

To estimate the biodegradability of bioplastics in the anaerobic fermentation plants operating at thermophilic temperature under slurry conditions, the Japan BioPlastics Association (JBPA) and Japan Bioindustry Associaion (JBA) started a standardization project with the National Institute of Advanced Industrial Science and Technology (AIST), Fuji-Tokoha University, and the University of Shizuoka. In this project, a new evaluating system (commercial name “MODA-B”) is used for measuring the biodegradability of bioplastics. In this system, the gas evolved from the experimental vessel is collected in a gas sampling bag, then the gas volume is measured using a syringe, and the biogas component is analyzed by gas chromatograph (GC). This method is more convenient for measuring the evolved biogas volume than using a pressure transducer or inverted graduated cylinder submerged in water. With this system, convenient measurement of plastic biodegradability not only at the thermophilic temperature, but also at the mesophilic temperature is expected under slurry conditions.

In this paper, we describe the new evaluating system MODA-B apparatus and the results of the anaerobic biodegradation of PLA as generally using bioplastics by the MODA-B apparatus at a thermophilic temperature (55 °C) and mesophilic temperature (35 °C) under aquatic conditions in which the TS were 2.07% (at 55 °C) and 2.24% (at 35 °C) in the anaerobic sludge.

## 2. Experimental Section

### 2.1. Plastic Sample

Cellulose powder of thin-layer chromatography grade with a particle size of less than 20 μm (Cellulose microcrystalline) was purchased from Merck (Germany). PLA (H-400, Mitsui Chemical. Co., Ltd., Japan) was kindly supplied by Mitsui Chemicals (Japan). The PLA powder was prepared by previously described methods [1,2]. The PLA powder with a particle size between 125 μm to 250 μm (Av. 180.7μm) was used.

### 2.2. Apparatus for Anaerobic Biodegradation Test

**Figure 1.**
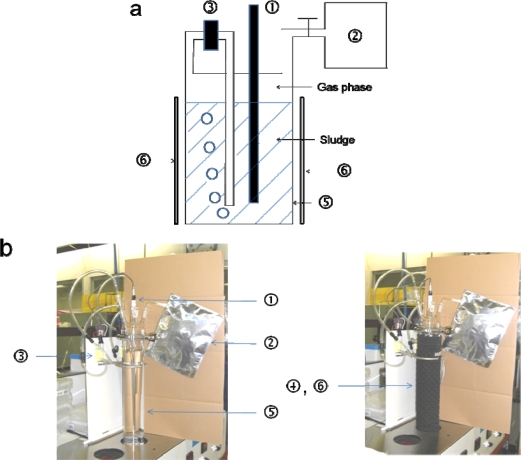
System of MODA-B apparatus. (a) Schematic of MODA-B; (b) Pictures of MODA-B. 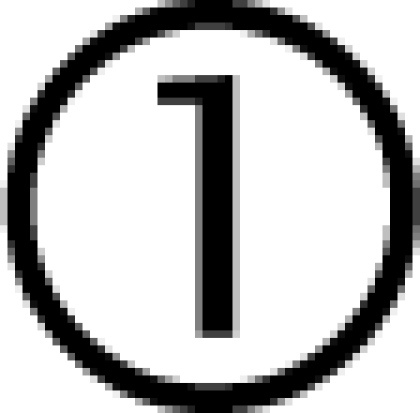
Temperature sensor, 
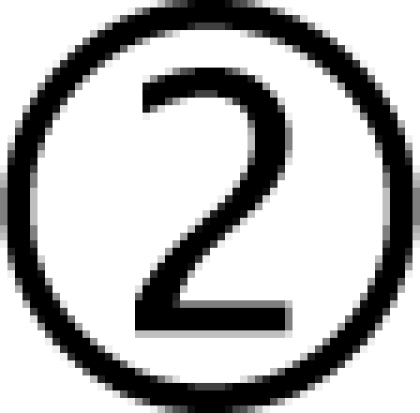
Gas sampling bag, 
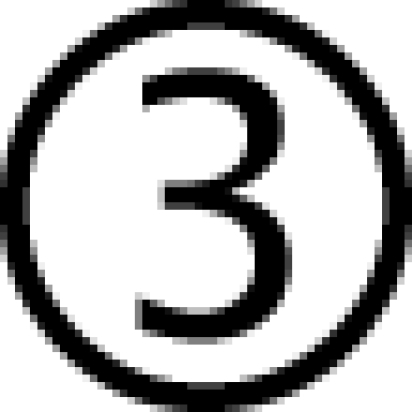
Pump (Gas phase in head space is exhausted from the bottom of the vessel by this pump), 
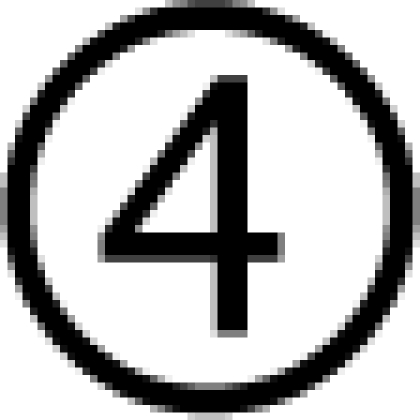
Surrounding envelope (there is a panel heater inside the black envelope), 
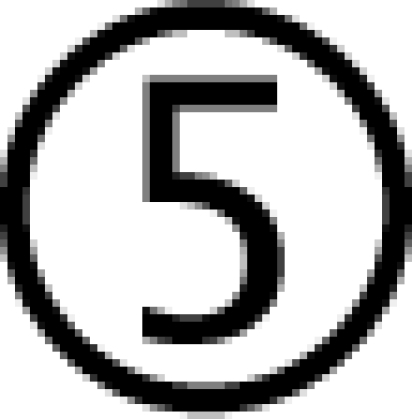
Test vessel, 
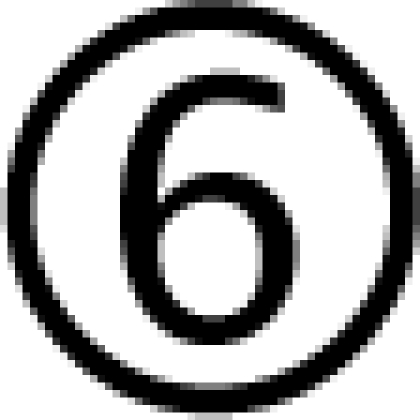
Panel heater.

The MODA-B apparatus (Yahata-Bussan Co., Ltd., Japan) is shown in Figure 1. The sludge and test material are sealed in the test vessel (1.5 L) and incubated at 55 °C. The sludge (1.4 L) is stirred by exhausting the gas phase from the upper head space to the bottom of the vessel using a gas pump, then mixing by gas bubble lifting. A panel sheet heater attached to the outside vessel with a surrounding cloth envelope maintained the temperature and control of the vessel temperature using a thermostat sensor in the middle of the vessel. The evolved biogas is collected in a gas sampling bag, which is an aluminum laminate with a four-layer structure (volume 2–5 L) and Teflon stopcock. The biogas volume in the bag was measured using a glass syringe (500 mL).

### 2.3. Procedure of Anaerobic Degradation Test

**Figure 2.**
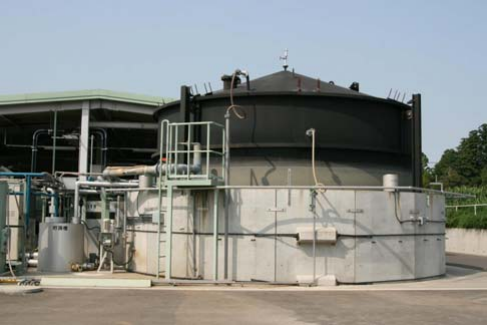
Fermentation tank in the Yamada Biomass Plant.

The Yamada Biomass Plant in Chiba Prefecture, Japan, operates an anaerobic fermentation tank (Figure 2) in which anaerobic fermentation mainly uses cow manure and vegetable waste as the feed stock at about 37 °C. The fermentation tank volume is 350 m^3^ and its retention time is 27 days. The sludge in this plant was used as the sludge for the anaerobic biodegradation test at 35 °C. There is no anaerobic fermentation plant operating at 55 °C around our laboratory, therefore, we made the sludge adapted at 55 °C. To make the sludge adapted at 55 °C, the sludge from the tank operating at around 37 °C in the Yamada Biomass Plant was preincubated at 55 °C [3].

**Figure 3.**
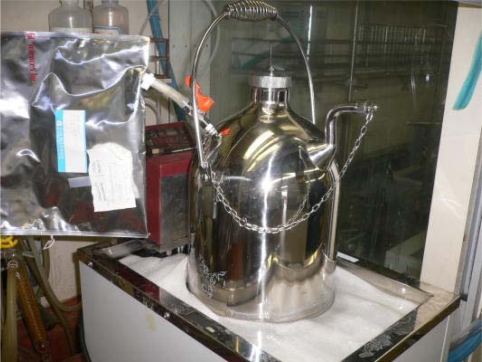
Preincubation of the sludge in stainless steel bottle.

The anaerobic sludge as indicated in Table 2 was obtained from the Yamada Biomass Plant. This sludge was poured into a stainless steel bottle (10 L) that contained a liquid pouring vent with a stainless steel cap and gas purge vent with a stopcock, which is generally used for volatile organic solvent, and carried to our laboratory. One bottle (10 L) was preincubated at 35 °C, and another bottle (10 L) was preincubated at 55 °C (Figure 3). At the beginning of the preincubation at 55 °C, 60 mL of the sludge, which previously degraded PLA in the plastic biodegradation test at 55 °C, was added as a seed sludge. The evolved gas from the sludge was collected in the gas sampling bag that was connected to the stainless steel bottle. When the H_2_S concentration of the evolved gas from the preincubated sludge was over 1,000–1,500 ppm, the gas phase was purged with air or N_2_. When the methane ratio was over 60% and the biogas evolution from the sludge decreased, the upper solution part of the sludge at 55 °C was transferred to a bucket. When the biogas evolution from the sludge decreased, the upper solution part of the sludge at 35 °C was transferred to a bucket. The total solid concentration, volatile solid, total organic carbon, and total nitrogen in the sludge solution part were determined by Eco Pro Research Co., Ltd. (Japan) as indicated in Table 2. The solution part of the preincubated sludge in the bucket was stirred, then 1.4 L of the sludge solution part was transferred to the vessel of the MODA-B apparatus mixing with the sample powder under ambient conditions. The incubation was done at 55 °C or 35 °C under sealed anaerobic conditions. The sludge was stirred for one minute a day by gas circulation from the upper head space to the bottom of the vessel. The evolving biogas was collected in a gas sampling bag (2–5 L) connected to the test vessel in the MODA-B apparatus.

### 2.4. Analysis of Evolved Biogas

The biogas volume in the gas sampling bag was measured using a glass syringe (500 mL). The H_2_S concentration in the bag was measured using a disposable detector tube. CO_2_ and CH_4_ in the bag were analyzed by a gas chromatograph (Shimadzu GC-8APT) equipped with a SHINCARBON ST column (2 m × 3 mm Φ, Shinwa Chemical Industries, Ltd., Japan) and thermal conductivity detector (80 °C initial temperature, 200 °C final temperature, 20 °C/min rate). The peak area ratio of (CH_4_/(CO_2_ + CH_4_)) obtained by GC was converted to a volume ratio of (V(CH_4_)/(V(CO_2_) + V(CH_4_)) using the calibration curve obtained by an experiment using standard gas of CO_2_ and CH_4_. The methane ratio (%) was calculated as 100 × V(CH_4_)/(V(CO_2_) + V(CH_4_)).

### 2.5. Inorganic Carbon Measurement in Sludge

Part of the evolved CO_2_ is dissolved in the sludge. It was postulated that the sludge was saturated with CO_2_ during the preincubation, so the sludge in the MODA-B apparatus was initially saturated. If the sludge in the test vessel contained more CO_2_ than the blank vessel during the test, the excess CO_2_ amount should be considered in the biodegradability calculation. Therefore, the CO_2_ amount in the sludge was measured at the end of the test.

Two mL of the sludge was diluted with 78 mL of deionized water, then 8 mL of a pH adjustment solution [7% citric acid (C_6_H_8_O_7_·H_2_O), 3.6% trisodium citrate dehydrate (C_6_H_5_Na_3_O_7_·2H_2_O), 8.24% NaCl] was added to the diluted sludge to lower the pH below 4. When the pH is below 4, bicarbonate and carbonate are converted to CO_2_. The CO_2_ concentration of the diluted sludge (below pH 4) was measured by a CO_2_ electrode (Ti-9004, Toko Kagaku Institute).

### 2.6. Calculation of Biodegradability

It is assumed that the gas sampling bag contained CO_2_, CH_4_ and saturated water vapor because water vapor is always evolving from the aquatic sludge solution. To determine the CO_2_ and CH_4_ volume in the evolved biogas, the water vapor volume should be subtracted. The CO_2_ and CH_4_ volume (V(CO_2_ + CH_4_)) was calculated using Equation (1)

(1) $$\text{V}({\text{CO}}_{2}+{\text{CH}}_{4})=((\text{P}(\text{atom})-\text{P}(\text{w}))/\text{P}(\text{atom}))\times \text{V}(\text{m})$$

where P(atom) is the atmospheric pressure in the laboratory at room temperature in hPa. P(w) is the vapor pressure of water at room temperature in hPa (from the water vapor pressure table in the CRC handbook [4]). V(m) is the measured gas volume in the gas sampling bag using a glass syringe in liters. This CO_2_ and CH_4_ volume was converted to the volume V(s) under standard conditions (at 273K, 1013 hPa) as indicated by Equation (2)

(2) $$\text{V}(\text{s})=(273/\text{T}(\text{r}))\times (\text{P}(\text{atom})/1013)\times \text{V}({\text{CO}}_{2}+{\text{CH}}_{4})$$

where T(r) is room temperature in Kelvin. V(s) is the CO_2_ and CH_4_ volume from the sample vessel under standard conditions in liters. The biodegradability (%) was calculated as indicated by Equation (3)

(3) Biodegradability (%)=((ΣV(s)−ΣV(b)+V(IC))/V(max))×100

where ΣV(s) is the total CO_2_ and CH_4_ volume from the sample vessel under standard conditions in liters. V(b) is the CO_2_ and CH_4_ volume from the blank vessel under standard conditions in liters. ΣV(b) is the total CO_2_ and CH_4_ volume from the blank vessel under standard conditions in liters. V(IC) is the CO_2_ volume dissolved in the sludge in excess of the blank value under standard conditions in liters ((CO_2_ volume dissolved in the sludge in test vessel) - (CO_2_ volume dissolved in the sludge in blank vessel)). V(max) is the maximum theoretical volume of the biogas (CO_2_ and CH_4_) evolved after complete biodegradation of the test material under standard conditions in liters. V(max) of the 10 g PLA ((C_3_H_4_O_2_)_n_) was calculated to be 9.3 L ((10/72) × 3 × 22.4). V(max) of the 10 g cellulose ((C_6_H_10_O_5_)_n_) was calculated to be 8.3 L ((10/162) × 6 × 22.4). In the above V(max) calculation, it is assumed that all the carbon in the material is converted to only CO_2_ and CH_4_ after complete biodegradation. The evolved (CO_2_ + CH_4_) volume is dependent on only the carbon amount regardless of the CO_2_ and CH_4_ ratio.

## 3. Results and Discussion

### 3.1. Preincubation of Sludge

**Figure 4.**
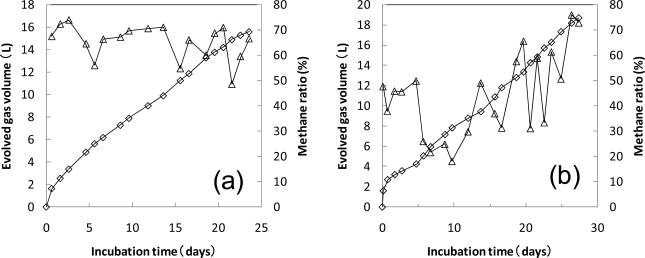
Evolved gas volume and methane ratio during preincubation of the sludges. The sludges were incubated at 35 °C (a) and 55 °C (b). ⋄; Evolved gas volume, Δ; methane ratio.

Figure 4 shows the preincubation of the sludge at 35 °C [Figure 4 (a)] and 55 °C [Figure 4 (b)]. For the preincubation at 35 °C, the biogas was constantly evolved from the start of preincubation and the methane ratio was about 60–70%. The methane ratio is generally about 60% under the regular anaerobic fermentation, then the sludge preincubated at 35 °C was thought to be normally fermenting. When the gas evolution decreased during the preincubation at 35 °C, the upper solution part of the sludge was used as the inoculum for the anaerobic biodegradation test of PLA at 35 °C.

On the other hand, preincubation at 55 °C was done by the method in which the sludge at 37 °C was incubated at 55 °C. Therefore, most of the microorganisms growing at 37 °C will be inactive or die at 55 °C, then the microorganisms that adapted to 55 °C were newly growing. In the first 5–6 days, the biogas evolution was at low levels. The methane ratio decreased in first 10 days. If the methane ratio is much lower than 60%, the anaerobic fermentation condition is not suitable for the degradation of organic materials. There was a transition period during the initial part of the preincubation at 55 °C after the temperature changed from 37 °C to 55 °C. The biogas evolution increased after 6 days and the methane ratio increased after 10 days, then it seemed that the microorganisms that adapted at 55 °C became gradually predominant. When the methane ratio was over 60% and the gas evolution decreased during the preincubation at 55 °C, the upper solution part of the sludge was used as the inoculum for the anaerobic biodegradation test of plastics at 55 °C.

The physico-chemical characteristics of the upper solution part of the sludge at the end of preincubation and the sludge in the tank of the Yamada Biomass Plant are shown in Table 2. The total solid concentrations of the sludges (upper solution part of the preincubated sludge) using the plastic degradation test were 2.24% (at 35 °C) and 2.07% (at 55 °C), down from 3.58% in the original sludge from the Yamada Biomass Plant because little sediment was included in the upper solution part. The total organic carbon amount in the sludge decreased from 0.98% to 0.38% (at 35 °C) and 0.37% (at 55 °C), because the remaining organic materials were consumed and sedimentary particles including the organic component were not included in the upper solution part.

### 3.2. Anaerobic Biodegradation Test of PLA by MODA-B

**Figure 5.**
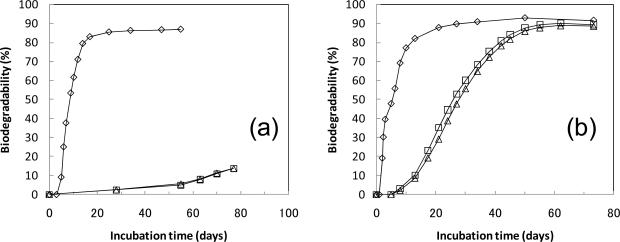
Provisional biodegradability in PLA anaerobic biodegradation test. The test vessels were incubated at 35 °C (a) and 55 °C (b). ⋄; cellulose 10g, Δ,□; PLA 10g. The biodegradabilities at each point were calculated as ((ΣV(s) – ΣV(b))/V(max)) × 100. The gas volumes from blank vessel were 0.03 (L) in 77 days (a) and 0.79 (L) in 73 days (b), measured at the end of the test. The gas volume from the blank vessel at each measuring point is calculated as ((incubation time)/77) × 0.03 (a), ((incubation time)/73) × 0.79 (b). The gas volume from the blank vessel at each measuring point is assumed to be the values when the volume from the blank vessel at the end of the test is proportionally divided by the incubation time.

Figure 5 shows the provisional anaerobic biodegradability calculated by the evolved biogas volume from the sludges containing cellulose or PLA powders at 35 °C and 55 °C using the MODA-B apparatus. Each vessel’s conditions of the MODA-B, such as the run, blank or sample, sample amount, incubation temperature, and dissolved CO_2_ amounts are indicated in Table 3. For the Figure 5 data, the excess dissolved CO_2_ volume in the sludge is not added. The biodegradability at each point was calculated as ((ΣV(s) − ΣV(b))/V(max)) × 100. The total evolved biogas volumes in the blank vessels were 0.03 L (77 days) at 35 °C and 0.79 L (73 days) at 55 °C under standard conditions (Table 3), when measured at the end of the tests. The gas volume from the blank vessel at each measuring point was calculated as

((incubation time)/77)×0.03 (Figure 5 (a)), ((incubation time)/73)×0.79 (Figure 5 (b)).

The gas volume from the blank vessel at each measuring point is assumed to be the values when the volume from the blank vessel at the end of the test is proportionally divided by the incubation time.

During the anaerobic biodegradation test at 35 °C [Figure 5 (a)], the cellulose was degraded 80% in 15 days. The gas evolution from the PLA vessel at 35 °C started in 55 days and biodegradation rate was very slow [about 2.9 (%/week)]. Therefore, the anaerobic biodegradation test from the PLA vessels at 35 °C was discontinued. During the anaerobic biodegradation test at 55 °C [Figure 5 (b)], the cellulose was degraded 80% in 13 days. PLA degraded about 60% in 30 days, about 80% in 40 days, and about 90% in 60 days. The dissolved CO_2_ amounts in each vessel are shown in Table 3. The final biodegradabilities (the excess dissolved CO_2_ volume in the sludge is added) are as follows: 87% (run 2, Cellulose 10 g), 93% (run 6, cellulose 10 g), 94% (run 7, PLA 10 g), and 92% (run 6, PLA 10 g).

**Figure 6.**
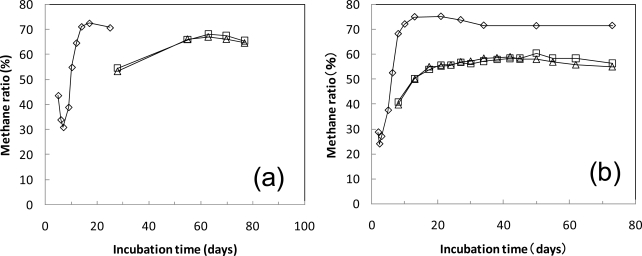
Methane ratio in PLA anaerobic biodegradation test. The test vessels were incubated at 35 °C (a) and 55 °C (b). ⋄; cellulose 10g, Δ,□; PLA 10g.

Figure 6 shows the methane ratio during the biodegradation of the cellulose and PLA powders in the sludge at 35 °C and 55 °C. During the beginning of the cellulose degradation, the methane ratio was low. As the cellulose degradation progressed, the methane ratio increased to a high value. The methane ratio in the total evolved biogas during the cellulose degradation was 47% at 35 °C and 50% at 55 °C. The methane ratio during PLA degradation at 35 °C was stable at about 60% after the gas evolution started (in 55 days). The methane ratio during PLA degradation at 55 °C was low during the first period in the initially low evolved biogas, then was stable between 50% and 60%. The methane ratio in the total evolved biogas during the PLA degradation was 55% and 56% at 55 °C.

**Figure 7.**
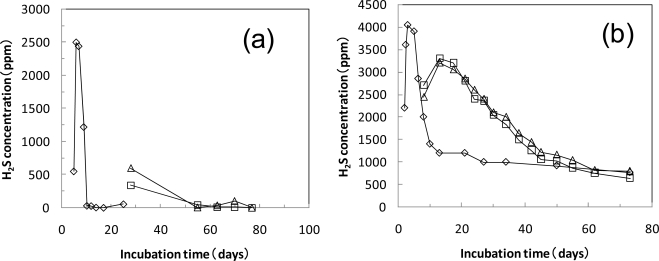
H_2_S concentration in PLA anaerobic biodegradation test. The test vessels were incubated at 35 °C (a) and 55 °C (b). ⋄; cellulose 10g, Δ,□; PLA 10g.

Figure 7 shows the H_2_S concentration during the anaerobic biodegradation of the cellulose and PLA powders. For the cellulose degradation at 35 °C, the H_2_S concentration was high at over 2,000 ppm during the initial degradation, but rapidly decreased as the degradation progressed. For the PLA degradation at 35 °C, the H_2_S concentrations were low after 55 days when the biogas evolution was observed. The H_2_S concentrations were high at over 2,000 ppm during the initial term at 55 °C. As the degradation progressed at 55 °C, the H_2_S concentration decreased.

In past studies, PCL and PLA showed a slight biodegradability under aquatic conditions at the mesophilic temperature [5–7]. PCL and PLA were not degraded when using the ISO 14853 method [5]. 7.6% of the PCL film was lost in the methane sludge after 10 weeks at 37 °C [6]. PLA was not degraded at all after 100 days using the ASTM D5210 method [7]. In our experiment, the PLA degradation was slow at 35 °C. Biogas evolution from the PLA vessels at 35 °C were observed in 55 days and the biodegradation rate was 2.9%/week. The anaerobic fermentation tank operating at 35 °C is thought to be unsuitable for the PLA degradation. For the high-solid test at the thermophilic temperature, PLA was degraded to 60% after 40 days at 52 °C using the ASTM D5511 method [8]. In our experiment, PLA degraded about 60% in 30 days, about 80% in 40 days, and about 90% in 60 days using the new evaluation system. PLA is thought to be degraded in the anaerobic fermentation tank at the thermophilic temperature under slurry conditions. However, the PLA degradation rate in a laboratory environment was slow compared to the general retention time (about 30 days) in the anaerobic fermentation tank.

## 4. Conclusions

In this study, we introduce a new method for evaluating the anaerobic biodegradation of plastics. This new method appears to be a convenient and effective one for estimation of the anaerobic degradation of bioplastics. Because the evolved biogas is collected in a gas sampling bag at atmospheric pressure with this system, therefore no use of a pressure transducer or inverted graduated cylinder submerged in water. With this system about 90% degradation of PLA powder was measured under the studied conditions at 55 °C.

## Figures and Tables

**Table 1.** Anaerobic biodegradation evaluation methods based on International Standards.

	**ISO 14853**	**ISO 15985**	**ASTM D5210**	**ASTM D5526**
Total solid concentration	0.1-0.3%	>20%	>0.1%	35, 45, 60%
Incubation temperature	35 ± 2 °C	52 ± 2 °C	35 ± 2 °C	35 ± 2 °C
Volume	0.1-1 L	>750 mL	100 mL	>800 g
pH	6.8–7.2	7.5–8.5		7.5–8.5
Sample amount	20–200 mg/L (as organic carbon)	20 g/vessel	Sufficient carbon content sample	sufficient carbon content sample
Inoculum	domestic sewage or laboratory-grown anaerobic sludge	household waste	well-operated anaerobic sludge	household waste

ISO14853: Plastics-determination of the ultimate anaerobic biodegradation of plastic materials in an aqueous system-Method by measurement of biogas production.

ISO15985: Plastics-determination of the ultimate anaerobic biodegradation and disintegration under high-solids anaerobic-digestion conditions-Method by analysis of released biogas.

ASTM D521: Standard test method for determining the anaerobic biodegradation of plastic materials in the presence of municipal sewage sludge.

ASTM D552: Standard test method for determining anaerobic biodegradation of plastic material under accelerated landfill conditions.

**Table 2.** Characterization of the sludge for anaerobic biodegradation test and original sludge.

	**Sludge in the tank of the Yamada Biomass Plant**	**35 °C**	**55 °C**
Total solid concentration (%)	3.58	2.24	2.07
Volatile solids (%)	2.03	1.11	1.03
Total organic carbon (%)	0.98	0.38	0.37
Total nitrogen (%)	0.30	0.24	0.24
C/N ratio	3	2	2
pH	8.3	8.3	8.5

**Table 3.** Dissolved CO_2_ amount in the sludge, biodegradability, pH and total methane ratio at the end of the anaerobic biodegradation of cellulose and poly(lactic acid) (PLA) powders.

**Run**	**Dissolved CO_2_ amount (mg) in sludge (1.4 L) in the vessel**	**Excess dissolved CO_2_ amount (-blank)**	**ΣV^1^ (L)**	**Biodegradability^2^ (%)**	**Total methane (%)**	**pH**
Incubation at 35 °C						
1 blank	13,890		0.03			8.0
2 Cellulose 10 g	14,000	110 mg (0.06 L)	7.22	87	47	8.0
3 PLA 10 g	15,290	1,400 mg (0.71 L)	1.30	21*		7.7
4 PLA 10 g	15,400	1,510 mg (0.77 L)	1.30	22*		7.8
Incubation at 55 °C						
5 blank	14,390		0.79			8.5
6 Cellulose 10 g	14,670	280 mg (0.14 L)	8.39	93	50	8.4
7 PLA 10 g	15,180	790 mg (0.40 L)	9.10	94	55	8.3
8 PLA 10 g	15,120	730 mg (0.37 L)	9.02	92	56	8.2

^1^ ΣV: total evolved CO_2_ and CH_4_ volume under standard conditions.

^2^ The biodegradability was calculated from gas volume evolved from sample vessel, gas volume evolved from blank vessel, and excess dissolved CO_2_ amount according to equation (3). V(max) of 10 g cellulose and PLA are 8.3 and 9.3 L, respectively.

* Biodegradation tests (runs 3 and 4) were discontinued. The biodegradability value is shown when the test was stopped.

## References

[1] Kunioka M Ninomiya F Funabashi M Biodegradation of poly(lactic acid) powder proposed as the reference test materials for the international standard of biodegradation evaluation methods Polym. Degrad. Stab 2006 91 1919 1928

[2] Funabashi M Ninomiya F Kunioka M Biodegradation of polycaprolactone powders proposed as reference test materials for international standard of biodegradation evaluation method J. Polym. Environ 2007 15 7 17

[3] Yagi H Ninomiya F Funabashi M Kunioka M Anaerobic biodegradation tests of poly (lactic acid) and polycaprolactone using new evaluation system for methane fermentation in anaerobic sludge Polym. Degrad. Stab 2009 94 1397 1404 10.3390/ijms10093824 PMC2769157 19865521

[4] CRC Handbook of Chemistry and Physics75th ed Lide DR Frederikse HPR CRC Press Boca Raton, FL, USA 1994 1995

[5] Massardier-Nageotte V Pestre C Cruard-Pradet T Bayard R Aerobic and anaerobic biodegradability of polymer films and physico-chemical characterization Polym. Degrad. Stab 2006 91 620 627

[6] Abou-Zeid DM Müller RJ Deckwer WD Degradation of natural and synthetic polyesters under anaerobic conditions J. Biotechnol 2001 86 113 126 11245900 10.1016/s0168-1656(00)00406-5

[7] Shin PK Kim MH Kim JM Biodegradability of degradable plastics exposed to anaerobic digested sludge and simulated landfill condition J. Environ. Polym. Degrad 1997 5 33 39

[8] Itävaara M Karjomaa S Selin JF Biodegradation of polylactide in aerobic and anaerobic thermophilic conditions Chemosphere 2002 46 879 885 11922068 10.1016/s0045-6535(01)00163-1

[9] Bouallagui H Touhami Y Cheikh RB Hamdi M Bioreactor performance in anaerobic digestion of fruit and vegetable wastes Process Biochem 2005 40 989 995

[10] Demirel B Yenigün O Two-phase anaerobic digestion processes: A review J. Chem. Technol. Biotechnol 2002 77 743 755

[11] Patel H Madamwar D Single and multichamber fixed film anaerobic reactors for biomethanation of acidic petrochemical wastewater-systems performance Process Biochem 2001 36 613 619

